# Inter- and Intrasexual Variation in Cuticular Hydrocarbons in *Trichrysis cyanea* (Linnaeus, 1758) (Hymenoptera: Chrysididae)

**DOI:** 10.3390/insects13020159

**Published:** 2022-02-01

**Authors:** David Fröhlich, Lukas Zangl, Günther Raspotnig, Stephan Koblmüller

**Affiliations:** 1 Institute of Biology, University of Graz, Universitätsplatz 2, 8010 Graz, Austria; lukas.zangl@uni-graz.at (L.Z.); guenther.raspotnig@uni-graz.at (G.R.); 2 Universalmuseum Joanneum, Studienzentrum Naturkunde, Weinzöttlstraße 16, 8045 Graz, Austria

**Keywords:** cytochrome c oxidase subunit I (COI), cuticular alkanes, ecological-chemotypes, intrasexual variation, chemical mimicry maturation, cuticular hydrocarbon polymorphism

## Abstract

**Simple Summary:**

The biology of many species of cuckoo wasps (Chrysididae) is largely unknown, except for, if at all, the host species of these brood parasites. Cuticular hydrocarbons (CHCs), which form a waxy layer on the body surface of insects, have been shown to be mimicked by host-specific cuckoo wasp species. We studied the CHC profiles of a rather generalist brood parasitic cuckoo wasp species, *Trichrysis cyanea*, in detail. We found sex-related differences in CHCs and three different chemotypes among females. Genetic data reject the possibility that these different chemotypes represent different (cryptic) species. The CHC polymorphism could be an adaptation for females to sneak into nesting holes of hosts with the different female chemotypes representing adaptations to a broader host range. However, since information on the CHC profiles of the hosts is missing, it remains unclear whether these different CHC profiles are used for chemical mimicry or are simply age-related, reflecting the females’ reproductive state.

**Abstract:**

Cuckoo wasps (Chrysididae, Hymenoptera) are known for their parasitoid or cleptoparasitic life histories. Indeed, the biology of only a few species has been studied in detail and often only little more is known than the host species. By mimicking their hosts’ cuticular hydrocarbon (CHC) profiles, species that parasitize single (or a few closely related) host species manage to deceive their hosts. However, the variability of the CHC profile in generalist cuckoo-wasp species is still unknown. Here, we used gas chromatography—mass spectrometry (GC-MS) and DNA barcoding to study intraspecific variation in cuticular hydrocarbons of one less host-specific species of cuckoo wasps, *Trichrysis cyanea*. Cuticular hydrocarbon (CHC) patterns were found to differ between males and females. Additionally, we found chemical polymorphism among females, which formed three distinct chemical subgroups characterized by different alkene patterns. A lack of divergence in the DNA barcoding region suggests that these different chemotypes do not represent cryptic species. Whether this intrasexual CHC-profile variation is an adaptation (mimicry) to different host species, or simply signaling the reproductive status, remains unclear.

## 1. Introduction

The cuticle of insects is covered by a thin layer of wax. These epicuticular lipids mainly consist of hydrocarbons with a typical length between 19 and 50 carbon atoms [1,2]. The three major chemical groups of cuticular hydrocarbons (CHCs) are n-alkanes, n-alkenes, as well as methyl branched alkanes. Different species have evolved with different CHC profiles. Notably, not only the number and identity of the CHC molecules on the cuticle varies among species, but also their relative abundance. These profiles are often species-specific in arthropods, such that CHCs are regarded as potentially useful markers in chemical taxonomy and systematics (e.g., [3]). Indeed, CHCs have been successfully used for resolving morphologically cryptic species complexes (e.g., [4,5]). CHCs have several functions in insects (and other arthropods). They prevent desiccation by building a hydrophobic barrier against water loss through the cuticula. They are also used for chemical communication, e.g., as pheromones, for mate recognition and mate choice, and they can impart information about gender, age, fertility, health and colony and caste affiliation [2,6,7]. In addition, CHCs serve as a barrier against microorganisms and play an important role in predator/parasite-prey interactions [7,8]. 

Parasitism is among the most successful evolutionary strategies, with around 40% of all known species having evolved parasitic lifestyles and a vast amount of species being parasitized [9,10]. Parasites optimize host utilization and hosts develop strategies to escape from parasites as much as possible, leading to corresponding adaptations in both hosts and parasites (e.g., [11,12,13]). Among parasitic insects, it has been suggested that 6.5–20% of all species may be parasitoid wasps [14], or more than 75% of all wasp species are parasitoids [15]. One family of wasps that includes species with parasitoid lifestyles is the family Chrysididae, also known as “cuckoo wasps”, with about 3000 species hitherto described. Their hosts are sawflies, aculeate wasps and bees, silk moths and walking sticks [16], but, in general, little is known about the biology of many species and usually, if at all, only the name of the host species is available [17,18,19]. Previous studies on the reproductive biology of chrysidid species implicate an important role of CHCs. Females of some species sneak into the nesting holes of their hosts to lay their eggs without being detected by their hosts. To be able to do so, many cuckoo wasps appear to mimic the CHC profiles of their hosts, but the exact strategies might differ among species, ranging from chemical insignificance, i.e., a much-reduced amount of CHCs as compared to the host [20], to weak mimicry, i.e., CHC profiles intermediate to those of different host species, in species parasitizing a few closely related host species [21], and strong mimicry of single host species [22]. Other species do not need any form of mimicry as the females open already closed nesting holes of their hosts [23] or lay their eggs into their hosts’ prey (e.g., [24]).

*Trichrysis cyanea* (Linnaeus, 1758) is a common cuckoo wasp species in the Palearctic region. It is a cleptoparasite that lays its eggs into the nests of different hosts and produces two to three generations per year. Known host species belong to the genera *Trypoxylon* (Crabronidae), *Auplopus* (Pompilidae) and *Deuteragenia* (Pompilidae), but other species (mainly from the families Crabronidae and Pompilidae) have also been reported as potential hosts [17,25]. Thus, this species has a rather broad host spectrum, but it is still unclear to what extent *T. cyanea* has adapted its life strategies to its various hosts. In the present study, using gas chromatography coupled to mass spectrometry (GC-MS) and DNA barcoding, we investigated CHC-profiles in *T. cyanea*. Specifically, we inferred (i) whether male and female *T. cyanea* have different CHC profiles, (ii) how variable CHC profiles are within sexes, and (iii) in case there are different CHC profiles, whether this indicates cryptic species. 

## 2. Materials and Methods

### 2.1. Sampling and Species Determination

In total, 24 females and 28 males of *T. cyanea* were collected at 14 locations in Eastern Austria in Styria, Lower Austria, Vienna and Burgenland from 2017 to 2018 (a map is provided in Figure S1). In most cases, at least two individuals were collected per sampling site, in a distance less than 250 m from each other. In addition, twelve individuals from six other species (*Chrysis fulgida* Linnaeus, 1761; *Chrysis inaequalis* Dahlbom, 1845; *Chrysis iris* Christ, 1791; *Chrysura laevigata* (Abeille de Perrin, 1879); *Chrysis gracillima* (Förster, 1853) and *Hedychridium roseum* (Rossi, 1790)) were collected and used as outgroup (Table S1). Specimens were identified following Linsenmaier [26].

### 2.2. Gas Chromatography—Mass Spectrometry

CHCs were extracted by whole body extraction of single individuals for 30 min in 70–150 µL of hexane, depending on the size of the specimens. Aliquots of the extracts (1.5 µL) were analyzed by GC-MS using a Trace gas chromatograph (Thermo, Vienna, Austria) equipped with a ZB-5 fused silica capillary column (Phenomenex, Aschaffenburg, Germany) coupled with a DCQ I mass spectrometer (Thermo, Vienna, Austria). Detailed information and a description of the settings can be found in [27]. CHCs were identified by diagnostic ions, indicating saturated or unsaturated hydrocarbon fragments (C_n_H_2n + 1_-ions; C_n_H_2n − 1_-ions), molecular weight (M^+^) and possible methyl branches. In case of unsaturated compounds, the position of double bonds was determined by dimethyl disulfide (DMDS) derivatization, following [28] with some modifications. In detail, we added 100 µL DMDS (dimethyl disulfide; Sigma Aldrich, Vienna, Austria) and 50 µL iodine solution to 20 µL of the extracts diluted with 80 µL of hexane. The reaction mixtures were kept at 50 °C overnight, then diluted with 500 µL of hexane and 500 µL of 5% sodium thiosulfate. After repeated extraction with hexane (500 µL, twice), the organic phase was dried over anhydrous magnesium sulfate and reduced to dryness with N_2_, re-dissolved in 30 µL hexane and analyzed by GC-MS. DMDS-derivatized alkenes were identified on the basis of the characteristic cleavage at the carbon-atom at the site of derivatization, leading to two intense ions that corresponded to the parts of the molecule at both sides of the double bond. In case of minute quantities, derivatives did not show all mass spectrometric characteristics, but could be assigned due to their retention time in other samples. Gas chromatographic retention indices (RI) were calculated using an alkane standard mixture and a calculated (median) retention time of extract compounds [29]. We were able to unambiguously identify all components with a relative abundance of more than 1% that occurred in at least 10% of the individual extracts and quantified these for statistical analysis. Isomers with lower amounts were considered if they could be identified unambiguously. The peak areas were calculated using Xcalibur 2.0.7™ (Thermo Fisher Scientific, Vienna, Austria) and the relative amounts (%) were calculated as peak area relative to the total area of all listed compounds. Statistical analyses were conducted with PAST 3.17 [30]. Non-metric multidimensional scaling (NMDS) based on Bray–Curtis dissimilarity [31] was performed to identify chemical clusters. One-way PERMANOVA [32] was used to test whether the differences between clusters were statistically significant.

### 2.3. DNA Extraction, DNA Barcoding and Genetic Analyses

After extracting the CHCs, one leg was cut off and stored in 250 μL of >99% ethanol at −20 °C. Genomic DNA was extracted following a rapid Chelex protocol [33]. The first part of the mitochondrial cytochrome c oxidase subunit 1 (COI) gene was amplified, purified and sequenced following [34]. The primers used for PCR and cycle sequencing were LCO1490 and HCO2198 [35], with an annealing temperature of 50 °C. Failed PCRs were repeated using the Phusion Flash High-Fidelity PCR Master Mix (Thermo Fisher Scientific) following the manufacturer’s instructions.

Nucleotide sequences were edited in MEGA version X [36] and checked by eye. The nucleotide sequences were aligned using the ClustalW algorithm implemented in MEGA and checked for mitochondrial pseudogenes (numts) by translating them into amino acid sequences and looking for internal stop codons. For phylogenetic tree inference, a maximum likelihood (ML) tree was inferred in PhyML 3.0 [37], applying the best-fitting substitution model (GTR + I + G) selected by the Smart Model Selection tool [38] based on the Bayesian Information Criterion (BIC), with 1000 bootstrap replicates to assess statistical branch support. Visualization of the ML tree was done in MEGA version 5 [39]. The nucleotide sequences were deposited on BOLD (BACHR001-21-BACHR0064-21) and GenBank (OM415512-OM415571).

## 3. Results

### 3.1. Cuticular Hydrocarbon Patterns (CHCs)

In total, 23 compounds were identified and used for further analyses. These compounds were found to be alkanes and alkenes, ranging from 21 to 29 carbon atoms (see Table 1). We were not able to separate 13-nonacosene and 14-nonacosene, which occurred only in males, as well as 9- and 10-heneicosene from one single male sample. From characteristic fragments of the DMDS-derivatives, however, the identity of the two contributing compounds for each peak could be concluded. For example, derivatized 13- and 14-nonacosene eluted within one peak, exhibiting a mixed spectrum with a molecular ion at *m/z* 500, and additional fragments from cleavage at position of double bonds at *m/z* 229 + *m/z* 271 (13-nonacosene), and *m/z* 243 + *m/z* 257 (14-nonacosene). As we were not able to calculate the relative amount of the single isomeric forms of these two mixtures separately, we treated them as a combined character in further analyses.

Furthermore, a few additional but inconsistently occurring compounds were found in the samples. These were hentriacontane (C31), hentriacontene (C31:1), and some methyl-branched hydrocarbons that occurred in traces in some of the extracts. The low amounts of these compounds allowed a tentative identification only. These compounds were not considered for statistics.

**Figure 1:**
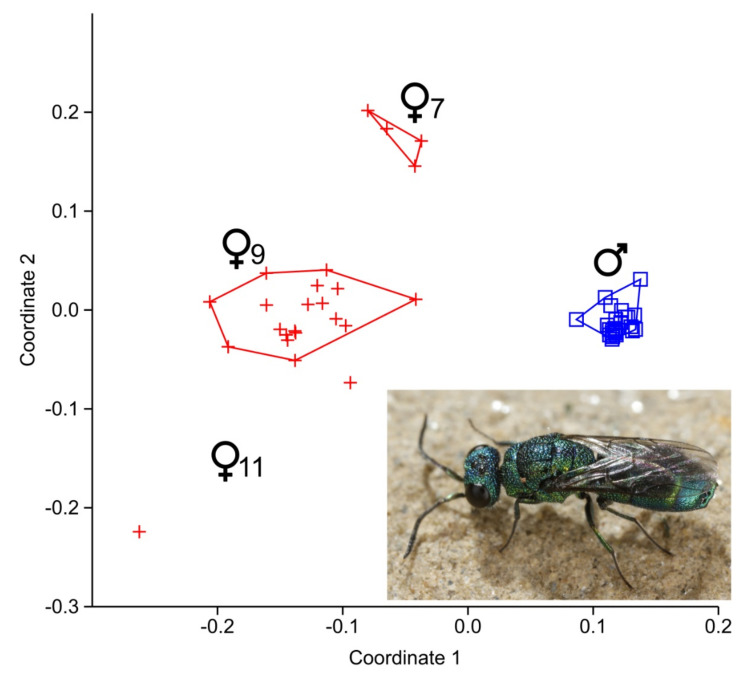
Non-metric multidimensional scaling (NMDS) based on Bray-Curtis distances of all individual cuticular hydrocarbon (CHC) profiles of *Trichrysis cyanea*. Males are indicated by blue squares, females by red crosses. Note the distinct female clusters (also see dendrogram in Figure S2). The different female clusters are named after the position of double-bonds of the main CHCs. The photo shows a living individual of *T. cyanea*. Photo: Gernot Kunz.

A comparison of individual CHC-patterns by NMDS clearly indicated chemical sexual dimorphism (Figure 1; *p* < 0.0001). While the male specimens cluster together, the females appear to be chemically more heterogeneous and form three distinct subclusters (Figure 1 and Figure S2). 

**Figure 2:**
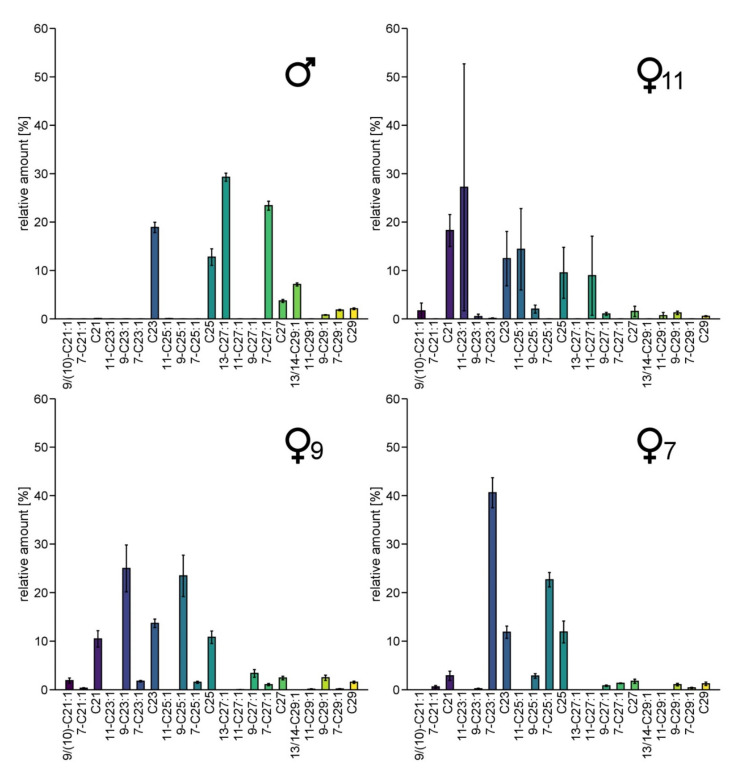
Average amount of CHCs in males and the three groups of females of *Trichrysis cyanea* that differ in their CHC profiles from each other. The female specimens were grouped regarding the most frequent double bond position. The relative amounts [%] are calculated as peak area relative to the total area of all listed compounds. Whiskers represent the standard error. Number of individuals per group: ♂: n = 25 ♀7: n = 4, ♀9: n = 18, ♀11: n = 2.

CHC-profiles of males showed large amounts of 13-heptacosene, 7-heptacosene, 13- and 14-nonacosene and 7-nonacosene (Figure 2). Females exhibited larger amounts of tricosene and pentacosene than males, with varying double bond positions, either in position 7, 9 or 11, respectively. According to the position of the double bond of the main alkenes, we identified three different female chemotypes, named ♀7, ♀9 and ♀11 (this terminology is also used in the figures). Only two ♀11 specimens are included in our dataset. Unlike the specimens of the other two clusters, their CHC-profiles show rather high variation in the relative abundance of particular CHCs, but as they were the only two female samples containing 11-C23:1 and 11-C25:1, we classified them as belonging to the same distinct subgroup. We were also able to detect 11-C27:1 not only in the two ♀11-females, but in traces (relative amount of 0.17–0.23%) in three other female samples. Thus, the different female subgroups are characterized not only by different components but also by different amounts of commonly shared substances. No correlation between the CHC-patterns and geography was observed, and all chemotypes were found at the sampling site with the largest sampling size (Figure S2).

### 3.2. DNA Barcoding

A total of 60 nucleotide sequences ranging from 541 to 657 base pairs (bp) in length were obtained, translating to a sequencing success rate of 94%. A deletion of three bp (position 345 to 347) was present in *T. cyanea*, *C. gracillima* and *C. laevigata.* All species represented by multiple samples were inferred as monophyletic entities in the phylogenetic analysis (Figure S3). We found little intraspecific variation in the COI nucleotide sequences of *T. cyanea* (pairwise distances of 0–0.7%; all 48 sequences are included in a single BIN (BOLD:AAH7935). Most *T. cyanea* individuals shared a single haplotype; four samples differed from this main haplotype by one substitution. These four specimens were all collected in Styria at three different sampling sites.

## 4. Discussion

In this study, we investigated CHC profiles of male and female *T. cyanea* and found a clear sexual dimorphism. In addition, we found three distinct CHC-profiles in females while males expressed a single profile with little variation. 

Sexually dimorphic CHC-profiles have already been reported for other chrysidids, namely *Chrysis pseudobrevitarsis* and *Chrysis parabrevitarsis* [5], and it is very likely that this is a general pattern in the family. Usually, both sexes of cuckoo wasp species inhabit the same habitat and, thus, abiotic factors are not expected to affect their CHC-profiles differently. Males and females may utilize different CHC compounds for different communication purposes, with CHC compounds of males probably mainly serving for mate recognition. In females, the CHC-inventory might be further shaped by the need to deceive their hosts. For example, females of *Hedychrum rutilans* mimic the CHCs of their host *Philanthus triangulum*, but they produce only low amounts of host-CHCs [22]. Furthermore, the amount of hydrocarbons on the cuticle of cleptoparasitic *H. rutilans* is five times less compared to the host, a phenomenon termed chemical insignificance [20]. Female *Chrysis mediata* Linsenmeier, 1951 and *Pseudochrysis neglecta* (Shuckard, 1837) are able to mimic different chemotypes of their host species *Odynerus spinipes* (Linnaeus, 1758) [23,40], whereas the chrysidid *Parnopes grandior* (Pallas, 1771) evolved a weak mimicry to multiple host species of the genus *Bembix,* mimicking only one single intermediate CHC-profile that includes major characteristics of all its hosts [21]. Comparably, the different chemotypes found in *T. cyanea* females may indicate an adaptation to different hosts. Common hosts of *T. cyanea* are species of the genera *Trypoxylon* (Crabronidae), *Auplopus* (Pompilidae) and *Deuteragenia* (Pompilidae) [25], but additional hosts (other crabronid and pompilid species) have also been reported [17]. Unfortunately, no data are available on host-CHCs to explicitly test which strategy of host deceit *T. cyanea* is following. 

Theoretically, the different CHC-profiles could have indicated the presence of cryptic species within the morphospecies *T. cyanea*, as has been shown for other chrysidids [5]. Our DNA barcoding data, however, strongly argue against cryptic diversity, unless divergence was very recent. Indeed, the genetic diversity in our *T. cyanea* samples was very low, which was unexpected given the generally high substitution rate of mitochondrial genes in Hymenoptera, and especially in Apocrita [41,42,43,44]. 

Distinctly different female chemotypes in *T. cyanea* may also reflect age-related differences, with CHC-modifications possibly occurring in different phases of maturation. Thus, different CHC-chemotypes of *T. cyanea* may characterize the cuticle chemistry of pre-reproductive, reproductive and post-reproductive (already mated) individuals. Pre-reproductive phases in insects can last between one day and several weeks (summarized in [45]). It has been shown that CHCs clearly correlate with reproductive status in some species [46,47], e.g., they are expressed in larger amounts at sexual maturity [45,48,49], and that they might be used as short-range contact pheromones [6,50]. *Trichrysis cyanea* produces multiple generations over a year, and well-founded data on CHC-modification during maturation would require an in-depth investigation of CHCs over a whole flight period.

## 5. Conclusions

In this study, we have shown that in the cuckoo wasp *Trichrysis cyanea*, CHC-profiles not only differ between the sexes but also among females. While sexual dimorphism can be explained by different communication purposes in males and females, the reasons underlying the evolution of distinct female chemotypes remain elusive. DNA barcoding results suggest that these female chemotypes do not represent distinct species, but whether these chemotypes are adaptations (mimicry) to different host species, or are simply signaling the reproductive status, remains unclear. To fully understand the complex function of the different CHC-profiles in *T. cyanea*, future studies will have to extend analyses of CHC-profiles also to the hosts and female *T. cyanea* of different and well identified maturation status. As *T. cyanea* is parasitizing a large number of host species from different families and produces multiple generations a year, this, however, will be a challenging endeavor. Nonetheless, such studies will provide important insights into how a generalist parasite has adapted to a broad host range and to what extent CHCs indeed play a role in this process. 

## Figures and Tables

**Table 1:** List of all components found and analyzed at the cuticula of male and female specimens of *T. cyanea*, with their retention index and abbreviation.

Name	Retention Index	Abbreviation
9- and 10-Heneicosene	2076	9/(10)-C21:1 ^1^
7-Heneicosene	2082	7-C21:1
Heneicosane	2100	C21
11-Tricosene	2274	11-C23:1
9-Tricosene	2277	9-C23:1
7-Tricosene	2284	7-C23:1
Tricosane	2301	C23
11-Pentacosene	2472	11-C25:1
9-Pentacosene	2477	9-C25:1
7-Pentacosene	2484	7-C25:1
Pentacosane	2500	C25
13-Heptacosene	2670	13-C27:1
11-Heptacosene	2671	11-C27:1
9-Heptacosene	2677	9-C27:1
7-Heptacosene	2685	7-C27:1
Heptacosane	2700	C27
13- and 14-Nonacosene	2868	13/14-C29:1 ^2^
11-Nonacosene	2871	11-C29:1
9-Nonacosene	2878	9-C29:1
7-Nonacosene	2886	7-C29:1
Nonacosane	2900	C29

^1^ A mixture of 10-C21:1 and 9-C21:1 occurred in sample DF028. ^2^ This mixture, not separable by GC, was found in all male individuals.

## Data Availability

The CHC data is available from the first author upon request. The DNA sequence data generated in this study are available on GenBank under the accession numbers listed in the “Material and methods” section and are also available on BOLD via the Process IDs BACHR001-21-BACHR0064-21.

## Supplementary Materials

The following are available online at https://www.mdpi.com/article/10.3390/insects13020159/s1, Table S1: Specimen details, Figure S1: map of sampling sites, Figure S2: Dendrogram of CHC-cluster analysis; Figure S3: Maximum likelihood tree of partial COI sequences.
