# Supplementary material for: Inter- and Intrasexual Variation in Cuticular Hydrocarbons in Trichrysis cyanea (Linnaeus, 1758) (Hymenoptera: Chrysididae)
Source: Insects. 2022 Feb 1;13(2):159. doi: 10.3390/insects13020159 (PMC8880203; doi:10.3390/insects13020159)
Supplement: Supplementary file 1 [file insects-13-00159-s001.zip › FigureS1_insects.pdf]

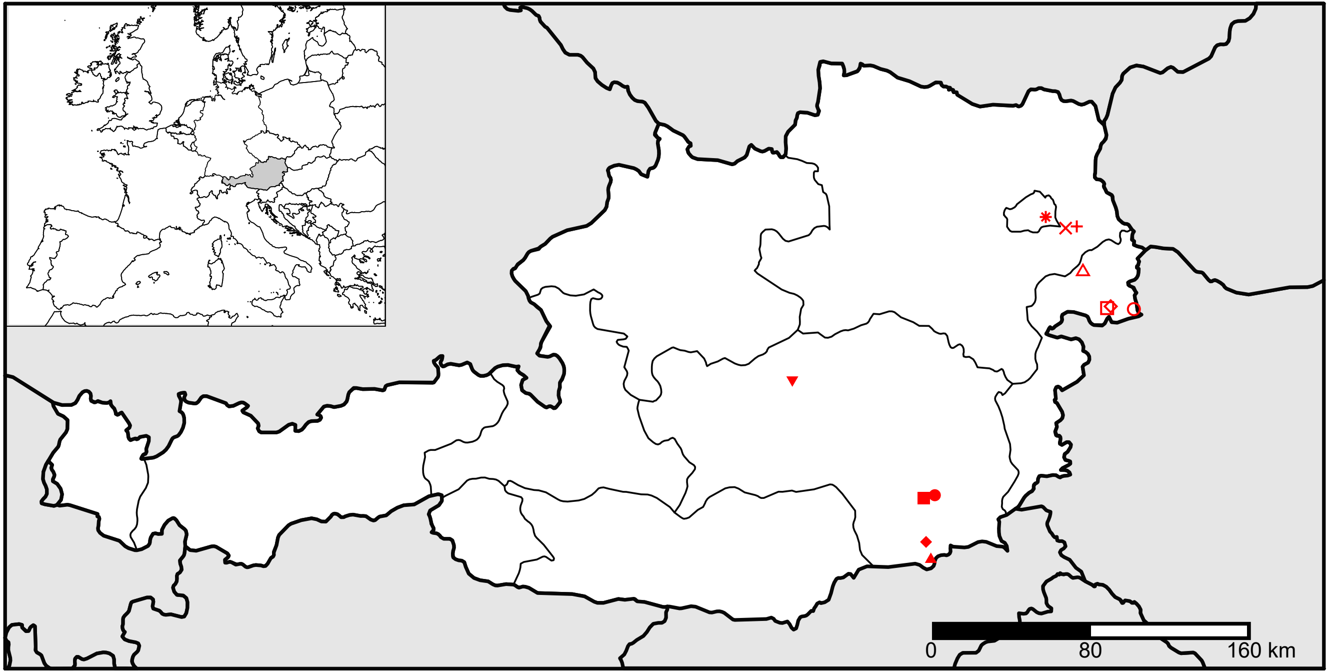

Figure S1. Map of the sampling sites for the present study in eastern Austria. Created with SimpleMappr (http://www.simplemappr.net/). The different symbols for each sampling site were used also in Figure S2. .
