# Supplementary material for: Inter- and Intrasexual Variation in Cuticular Hydrocarbons in Trichrysis cyanea (Linnaeus, 1758) (Hymenoptera: Chrysididae)
Source: Insects. 2022 Feb 1;13(2):159. doi: 10.3390/insects13020159 (PMC8880203; doi:10.3390/insects13020159)
Supplement: Supplementary file 1 [file insects-13-00159-s001.zip › FigureS2_insects.pdf]

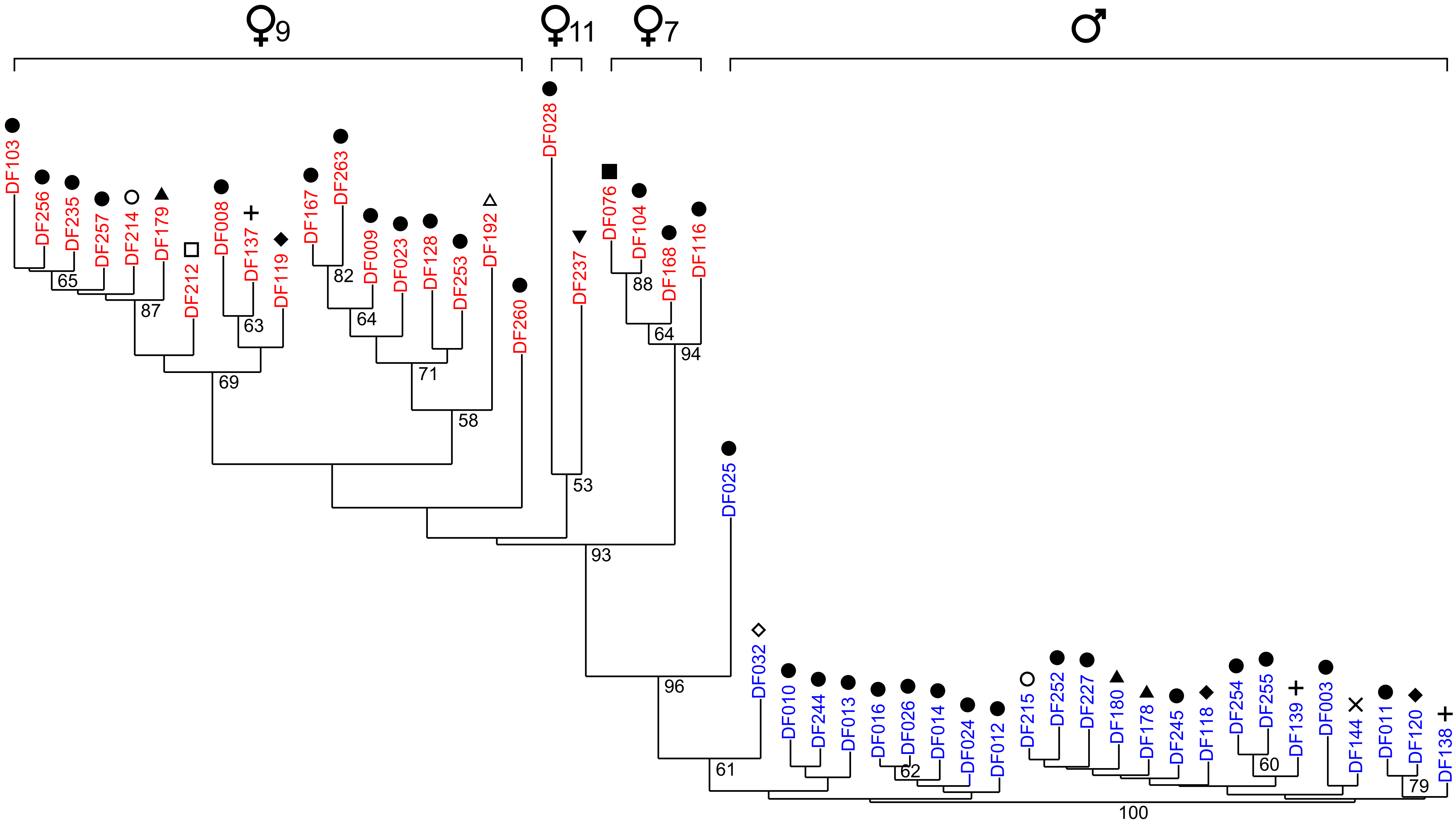

Figure S2. Cluster analysis using neighbour joining (similarity index: Bray-Curtis) were performed using PAST 3.17. Numbers at nodes indicate bootstrap support (1000 replicates, only values >50 are shown). The different female clusters are named after the position of double-bonds of the main CHCs. The symbols next to the sample IDs were used also in Figure S1 to distinguish between the different sampling sites.
