# Supplementary material for: Inter- and Intrasexual Variation in Cuticular Hydrocarbons in Trichrysis cyanea (Linnaeus, 1758) (Hymenoptera: Chrysididae)
Source: Insects. 2022 Feb 1;13(2):159. doi: 10.3390/insects13020159 (PMC8880203; doi:10.3390/insects13020159)
Supplement: Supplementary file 1 [file insects-13-00159-s001.zip › FigureS3_insects.pdf]

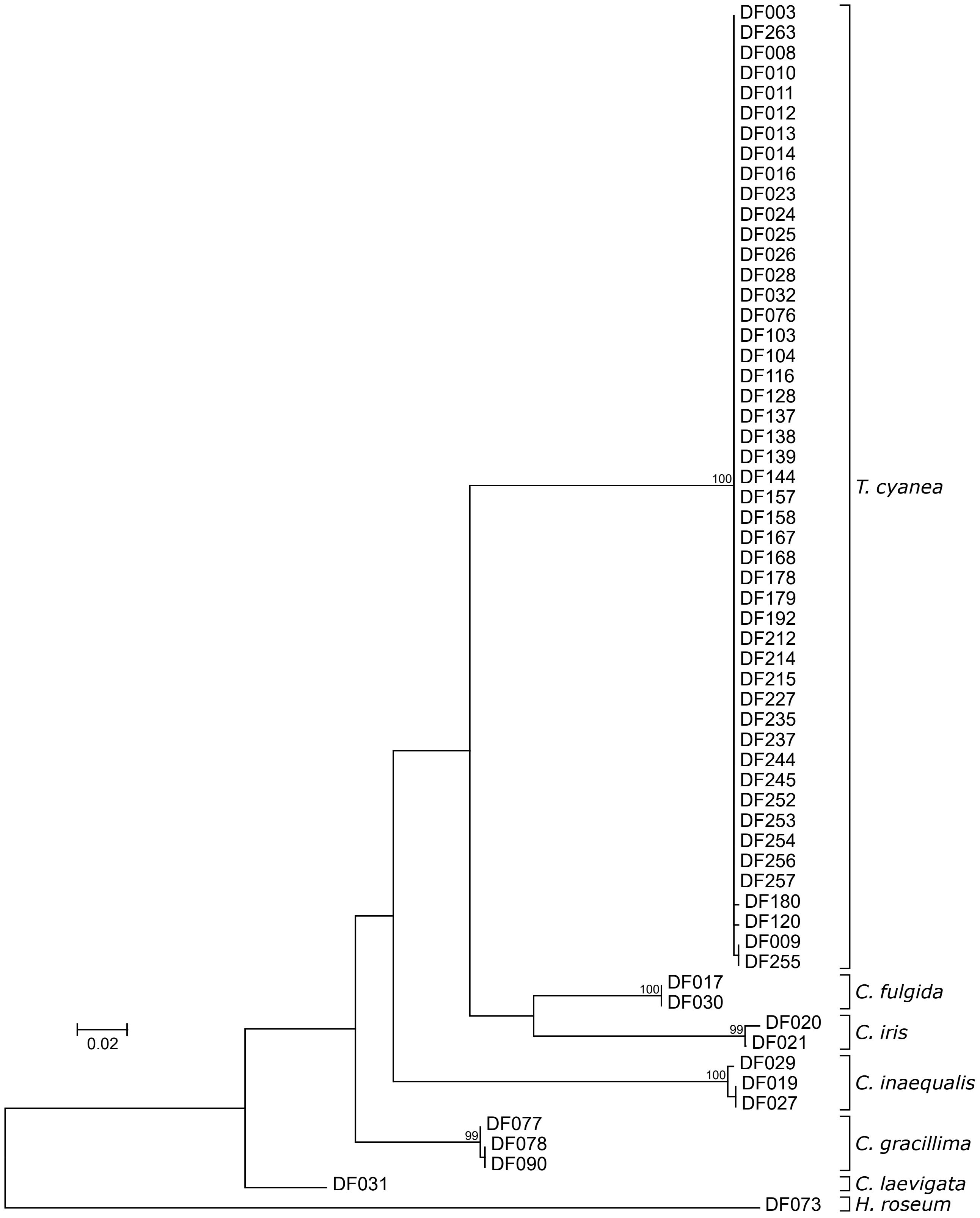

Figure S3. Maximum likelihood tree based on partial COI sequences of the collected specimens. Numbers at nodes indicate bootstrap support (only values >50 are shown).
